# Stakeholder Perspectives on mHealth Technologies to Prevent Sitting-Acquired Pressure Injuries in Long-Term Care Facilities: Mixed Methods Study

**DOI:** 10.2196/59590

**Published:** 2025-09-17

**Authors:** Tamara Vos-Draper, Erin Vinoski Thomas, Emily Graybill, Melissa Morrow, Kathleen A Jordan, Pamela R Manley, Sharon E Sonenblum

**Affiliations:** 1 Program of Occupational Therapy College of Pharmacy University of Minnesota Minneapolis, MN United States; 2 Center for Leadership in Disability School of Public Health Georgia State University Atlanta, GA United States; 3 School of Health Professions Center for Health Promotion, Performance, and Rehabilitation Research University of Texas Medical Branch Galveston, TX United States; 4 Nell Hodgson Woodruff School of Nursing Emory University Atlanta, GA United States; 5 School of Public Health Georgia State University Georgia State University Atlanta, GA United States

**Keywords:** pressure injuries, Alzheimer disease, dementia, wheelchair use, user-centered design, long-term care, prevention, assistive technology, monitoring, feedback

## Abstract

**Background:**

Adults with Alzheimer disease (AD) or Alzheimer disease and related dementias (ADRD) who require a wheelchair to accommodate disease-associated decline in mobility are at elevated risk for pressure injuries. More than half of residents in long-term care (LTC) facilities in the United States experience AD or ADRD. In LTC facilities, bed-based technologies exist to facilitate pressure injury prevention efforts, but similar technologies have not yet been widely evaluated to address sitting-related pressure injuries.

**Objective:**

This study aimed to determine preliminary design inputs from care providers for technology to address sitting-related pressure injury prevention in LTC settings. Specifically, we sought to (1) understand the types and use of sitting-related equipment used in LTC for residents with AD or ADRD, (2) identify challenges faced by nurses and other caregivers when repositioning seated residents, and (3) understand care provider preferences for features of future sitting-related feedback technologies designed to facilitate effective and timely repositioning.

**Methods:**

Surveys (n=30) and semistructured interviews (n=9) of administrative and direct care providers in LTC facilities were administered. Survey results were summarized, and we used thematic qualitative analysis of interview responses to develop themes around challenges experienced by care providers and their perceptions about how technologies could facilitate the prevention of sitting-related pressure injuries.

**Results:**

Survey respondents endorsed using many sitting surfaces for LTC residents with memory loss, such as padded reclining chairs, bedside or dining chairs, and wheelchairs with cushions. All indicated that shared equipment is provided by the facility, and 43% of respondents reported having access to a seating specialist at their facility. Sitting time was typically up to 12 hours per day. Themes related to pressure injury prevention in the LTC context, specific to those with memory loss, included (1) barriers to repositioning seated residents vary with the degree of memory loss, (2) care providers are aware of guidelines and policies around the 2-hour repositioning schedule, and (3) care providers are interested in technologies that have relative value over added burden. Care providers expressed interest in mobile health (mHealth) technologies that provide automatic repositioning in later stages of memory loss, delivery of cues for residents with mild memory loss to encourage independent repositioning, and tools to monitor resident sitting and pressure-related outcomes.

**Conclusions:**

These findings highlight the complexity of addressing the repositioning needs of seated LTC residents with AD or ADRD using mHealth technologies due to changes as the disease progresses. mHealth technologies should encourage more independence by residents experiencing milder memory loss, with increasing automaticity in repositioning residents in later stages. Both approaches could potentially minimize care provider burden in repositioning seated residents throughout the day. Design, development, and implementation of technologies should carefully weigh benefit versus burden to care providers and residents and continue to engage with them for feedback as development progresses.

## Introduction

### Background

Pressure injuries in long-term care (LTC) settings occur at high rates (3.4%-32.4%), and people with mobility decline and memory loss related to Alzheimer disease (AD) or Alzheimer disease and related dementias (ADRD) are among the populations at highest risk [1,2]. Furthermore, 58% of LTC facility residents in the United States have AD or ADRD [3]. The International Pressure Injury Prevention Guidelines [4] recommend repositioning at least every 2 hours in bed and while sitting to reduce the impact of prolonged pressure, which contributes to skin and tissue damage [4]. Health care facilities in the United States are motivated to reduce the prevalence of pressure injuries to meet standards of care and because their payments can be reduced if pressure injuries develop during the care they provide [5]. In the United States, the Centers for Medicare and Medicaid require systematic reporting of pressure injuries in LTC facilities, providing motivation to implement strategies to minimize their occurrence. To facilitate adherence to turning residents at least every 2 hours in bed, some facilities have implemented bed-based technologies that either automatically adjust the surface to manage pressure or provide auditory or visual feedback to caregivers [6-8]. However, bed-based technologies do not address sitting-related pressure from wheelchair use. As residents with AD or ADRD experience increasing mobility loss, they sit for longer periods, putting them at high risk for developing pressure injuries.

While some mobile health (mHealth) app options are now available to wheelchair users that provide cues or alerts to manage pressure, these technologies target independent wheelchair users living in the community [9,10]. Little is known about the use of monitoring or feedback technologies to facilitate caregiver efforts to reposition seated residents in residential care centers. Technologies to facilitate caregiving in the AD and ADRD population tend to focus more on case management, caregiver support, care pathways, or monitoring and tracking of the person with AD or ADRD for safety related to falls or wandering, and do not specifically address pressure injury risk [11,12]. This study is part of a multidimensional project spanning the development and usability of 2 mHealth systems that monitor and provide real-time feedback with the goal of increasing in-seat movement to prevent sitting-acquired pressure injuries [13-15]. One type of feedback under development focuses on a display showing pressure distribution at the seat-cushion interface and uses cues to remind the user to redistribute pressure throughout the day [13]. Pressure injury prevention is mutifaceted [4] and these technologies target user-driven actions to mitigate pressure at the seat interface. Pressure mapping at the seat interface is commonly used by occupational therapists (OTs) and physical therapists (PTs) during seating assessments and provides color-based depictions of the magnitude of pressure between a person and their sitting surface across a grid of at least 256 sensing areas [16]. The display of pressure distribution appears similar to a weather map and depicts areas of high pressure with yellow or red colors versus blue or green for areas of lower pressure. The development process for enhancing these mHealth systems has incorporated user-centered design [17,18] and their development was guided by behavior change frameworks [19].

The long-term goal of this project is to potentially adapt these mHealth systems for caregiver use in a setting where pressure injuries continue to be prevalent and to engage key stakeholders early in the design process to better understand their needs. Key stakeholders include LTC care providers who might have a direct impact on sitting, repositioning, and managing pressure injury prevention, such as certified nursing assistants (CNAs) and other nursing staff, and OTs and PTs. Findings from this research will identify important design criteria for developing and implementing such systems in an LTC setting, specifically to address the unique needs of residents with AD or ADRD.

### Research Questions

Research questions centered on understanding caregiver perspectives on pressure injury prevention during sitting including the use of feedback technologies:

1. In LTC settings, what factors impact the provision of care for preventing pressure injuries in wheelchair users with AD or ADRD?
2. What are direct care provider perceptions about the use of feedback technologies to facilitate repositioning residents in their wheelchairs?
3. What considerations are there for the design and development of wheelchair-based feedback technologies for caregiver use in the LTC setting?

## Methods

This cross-sectional study included a combination of survey and semistructured interviews of a small group of stakeholders who provide or oversee care in LTC settings. Stakeholders included nurses, CNAs, social workers, administrators, OTs, and PTs.

### Ethical Considerations

The study activities were reviewed and received approval from the appropriate institutional review boards (IRBs) at Georgia State (H22270), Georgia Institute of Technology (H21280), and the University of Minnesota (STUDY00016896). Each IRB determined that while the study qualified for exempt status, it met all institutional ethical standards and complied with relevant federal regulations for conducting the surveys and interviews. The 21-item checklist for the Standards for Reporting Qualitative Research was used for reporting guidance [20]. Informed consent was waived for respondents, and an information sheet describing the study and how data would be deidentified and used was provided and agreed to by participants before completing the survey or interview. All participant identifying information was stored in secure IRB-approved locations, accessible only by approved study staff. Data were deidentified before analysis and reporting of results. Survey participants were not compensated. Interview participants received compensation for their time.

### Survey Data Collection

#### Design and Administration

Two separate surveys were developed for this study. The first (survey A) was developed by researchers with expertise in wheelchair seating and pressure injury prevention from the Georgia Institute of Technology and Georgia State University, in collaboration with the University of Minnesota. The survey questions related to seating and mobility equipment used by residents in LTC, including those with AD or ADRD. The web-based survey was distributed via a web link using Qualtrics, a survey platform. Participants were asked to respond to questions related to characteristics of residents and care providers in their LTC facilities. For full details, refer to survey A in Multimedia Appendix 1.

The second survey (survey B) was developed by study team members with expertise in wheelchair seating and pressure injury prevention at the University of Minnesota in collaboration with partners at Georgia Institute of Technology, following the administration of survey A. The questions from survey A were refined to gather additional information about knowledge of feedback technologies for preventing pressure injuries, specifically related to awareness and receptiveness to various wheelchair-based monitoring technologies. Survey B included questions about which technologies the respondents were familiar with, which they thought might be most helpful to caregivers in preventing pressure injuries, and which technologies they would use if available. Survey B was administered using the University of Minnesota’s Research Electronic Data Capture system. Research Electronic Data Capture is a secure, web-based software platform designed to support data capture for research studies [21]. See survey B in Multimedia Appendix 1 for full details about the items in the survey, including annotations indicating where the survey differed from survey A.

#### Survey Participants

Survey A was made available to all LTC facilities in the state of Georgia, United States, that provide care to adults with AD or ADRD. The recruitment flyer for survey A was emailed individually to an administrator in all LTC facilities that provide care to adults with AD or ADRD. A follow-up phone call was made to each facility to confirm receipt of the survey link. An invitation to complete survey B was distributed by email to a convenience sample of rehabilitation care providers in LTC centers in Minnesota, United States, that were affiliated with the occupational therapy program as education sites. A follow-up email was sent after 2 weeks in an attempt to improve the response rate. Respondents were not offered remuneration for either survey A or B.

#### Survey Data Analysis

Survey responses and demographic variables for both surveys A and B were summarized using descriptive statistics for each to report the frequency of responses. Because there was no experimental design requiring inferential comparisons, descriptive analysis was the most appropriate to use.

### Interview Data Collection

#### Participants

Interview participants were recruited from LTC facilities that provide care to adults with AD or ADRD within a 90-minute radius of a large metropolitan area. The targeted LTC facilities were provided with a recruitment flyer by email and in person, along with a follow-up phone call to confirm receipt of the recruitment flyer. The sampling frame included direct care providers (nurses and CNAs), members from the administration, and other stakeholders, such as OTs and PTs, who are directly involved in the care of persons with AD or ADRD who use wheelchairs and live in skilled care facilities. The inclusion criteria for prospective participants are presented in Textbox 1.

**Inclusion criteria.**

**Inclusion criteria**

- Aged ≥18 years
- Work directly with patients who have Alzheimer disease or Alzheimer disease and related dementia or are a key stakeholder that may interact with the patients or systems
- Work at a skilled care facility or other facility that provides regular care to patients with Alzheimer disease or Alzheimer disease and related dementia and who use wheelchairs

For participating in the study, interviewees received a US $50 gift card, and study sites were compensated with a US $250 gift card for their involvement.

#### Interview Questions

Semistructured interviews were conducted to identify and understand the gaps in necessary care related to pressure injury prevention for wheelchair users with AD or ADRD in LTC. During interviews, participants were asked to discuss their respective roles, describe a typical workday, state knowledge of their employer’s existing weight shift policies, as well as to provide feedback on the utility and feasibility of implementing technologies designed to reduce the incidence of pressure injuries. The interview questions were developed by the research team to probe perspectives around the use of mHealth technologies specifically related to pressure injury prevention and can be reviewed in Multimedia Appendix 1.

To solicit feedback on the feasibility of personal-use technologies to prevent pressure injuries, interview participants were shown 2 short videos. The videos demonstrated 2 different in-seat tracking and pressure management systems that are commercially available. The systems shown were the Sensoria Mat (Sensoria Health Inc) and the Sensomative Wheelchair (Sensomative GmbH), both thin 40 cm × 40 cm fabric mats with embedded force sensors that can be placed under or inside of the wheelchair cushion and communicate with their respective apps to provide feedback about weight shifts with the intent to increase body movement and weight shifting. These novel, personal-use technologies were not originally designed for wheelchair users living with ADRD but may offer features that could be useful for this population. The videos were specifically designed to provide examples of the types of technologies that are available for the general wheelchair-user population, rather than to elicit feedback only on these specific products.

#### Interview Data Analysis

The interview data were recorded, transcribed verbatim, and then analyzed using a thematic analysis approach. Thematic analysis is a qualitative technique that identifies patterns in raw interview data with the aim of developing overarching themes [22]. To store, manage, and analyze the interview data, we used NVivo, a qualitative software platform by QSR International.

The first step in our thematic analysis was data familiarization. Each interview transcript was perused several times for familiarization, as well as to observe any meanings and patterns in the data. During data familiarization, analytic memos were maintained in NVivo to reflect on participant statements and meanings. Next, open coding, the process of creating initial codes, was completed. An example of an initial code was *prevention*, a code that refers to participant descriptions of any response, effort, intervention, or resource aimed at preventing pressure injuries (eg, weight shifts). The prevention code also referred to strategies staff used to assist residents in keeping their skin healthy (eg, skin creams).

After the open coding process, a codebook containing all the codes inductively created in step 2 was developed. Codes that generated the greatest number of references (ie, chunks of coded interview data) were highlighted. For instance, the code *prevention* contained 162 references. See Multimedia Appendix 1 for an illustration of the codebook.

The third step in our thematic analysis was to create in vivo codes using NVivo. In vivo codes are excerpts reflecting the participants’ own words. The excerpt, “I usually try to shift them every two hours,” is an example of an in vivo code. In our thematic analysis, step 3 resulted in nearly 600 in vivo codes. The fourth step was to associate select in vivo codes—codes that reflect a recurring pattern in the data—with the initial codes we created in step 2. Step 4 was a process of grouping or merging recurring patterns into themes.

## Results

### Survey Responses

There were a total of 30 respondents between survey A (n=22) and survey B (n=8). Demographic characteristics include respondents’ role in their facility, experience providing care to adults with AD or ADRD, their age, race, gender, and caregiver-to-resident ratio in the facility they work in (Table 1). Survey A included administrators, certified nursing and personal care assistants, and nurses, while survey B included primarily rehabilitation therapy staff and 1 nurse. All respondents of both surveys had >3 years of experience, and most respondents on survey A (n=13, 59%) and survey B (n=6, 75%) had at least 11 years of experience. All participants of the surveys were aged >30 years and predominantly female. The caregiver-to-resident ratio was similar between groups, with most participants of both surveys indicating a ratio of 1:6 to 1:10 in their facilities.

**Table 1:** Characteristics of survey and interview participants from long-term care facilities.

Characteristics		Survey A (n=22), n (%)^a^	Survey B (n=8), n (%)^b^	Interviews (n=9), n (%)
**Role**				
	Certified nursing assistant	2 (11)	0 (0)	5 (56)
	Personal care assistant	2 (11)	0 (0)	0 (0)
	Registered nurse	2 (11)	1 (12)	0 (0)
	Licensed practical nurse	2 (11)	0 (0)	0 (0)
	Administrator	8 (42)	0 (0)	3 (33)
	Operational director	1 (5)	0 (0)	0 (0)
	Social worker	0 (0)	0 (0)	1 (11)
	Occupational therapist or physical therapist	0 (0)	7 (88)	0 (0)
**Experience (y) in Alzheimer disease or Alzheimer and related disease care**				
	3-5	3 (16)	0 (0)	N/A^b^
	6-10	3 (16)	2 (25)	N/A
	≥11	13 (68)	6 (75)	N/A
**Age (y) of participants**				
	30-39	4 (24)	5 (62)	N/A
	40-49	2 (12)	2 (25)	N/A
	50-59	8 (47)	0 (0)	N/A
	≥60	3 (18)	1 (12)	N/A
**Race**				
	Black or African American	3 (18)	0 (0)	N/A
	Multirace	1 (6)	0 (0)	N/A
	White	12 (71)	8 (100)	N/A
**Sex**				
	Female	14 (82)	7 (88)	N/A
	Male	3 (18)	1 (12)	N/A
**Caregiver-to-resident ratio in the facility**				
	1:6-1:10	9 (47)	7 (88)	N/A
	1:11-1:15	8 (42)	1 (12)	N/A
	>1:15	2 (11)	0 (0)	N/A

^a^n=19 for role, experience, and caregiver-to-resident ratio and n=17 for age, race, and sex.

^b^Not applicable.

The typical daily sitting time for the residents in their facilities and the types of surfaces used are reported in Tables 2 and 3, respectively. The 22 responses from survey A indicate that residents in their facilities sit for 0 to 6 hours per day in a wheelchair (n=13, 59%) and for 0 to 6 hours per day on other surfaces (n=15, 68%), resulting in the potential cumulative of up to 12 hours per day for some residents who may be included in both groups. About half (n=11, 50%) of the responses indicated that residents sit 7 to 12 hours or >12 hours per day.

**Table 2:** Care provider response for typical daily sitting time for long-term care residents with Alzheimer disease or Alzheimer disease and related dementias.

Resident characteristic	Sitting for 0-6 h, n (%)	Sitting for 7-12 h, n (%)	Sitting for >12 h, n (%)
All residents in wheelchairs (n=22)	13 (59)	7 (32)	4 (18)
All residents on other surfaces (n=22)	15 (68)	2 (9)	3 (14)
Mild memory loss (n=8)	2 (25)	4 (50)	2 (25)
Moderate memory loss (n=8)	0 (0)	7 (88)	1 (12)
Advanced memory loss (n=8)	1 (12)	5 (62)	2 (25)

**Table 3:** Types of sitting surfaces used in long-term care facilities for residents with Alzheimer disease and Alzheimer disease and related dementias, as reported by care providers, and methods of procurement.

Sitting surface characteristics			Survey A (n=22), n (%)		Survey B (n=8), n (%)
**Nonwheelchair sitting surfaces^a^**					
	Geriatric “geri” chair	11 (50)		6 (75)	
	Recliner	9 (45)		8 (100)	
	Nonreclining bedside chair	0 (0)		5 (62)	
	Dining room chair	6 (30)		0 (0)	
	Broda chair	3 (15)		0 (0)	
	Sofa	2 (10)		0 (0)	
	Glider or lounge chair	1 (5)		0 (0)	
**Wheelchair-based equipment^a^**					
	Wheelchair (all types)	22 (100)		0 (0)	
	Manual wheelchair	0 (0)		8 (100)	
	Power wheelchair	0 (0)		7 (88)	
	Power scooter	0 (0)		4 (50)	
**Mobility device provision^b^**					
	Personal purchase	17 (77)		8 (100)	
	Facility owned (shared)	5 (23)		8 (100)	
**Seat cushions^a^**					
	Air filled	7 (32)		7 (88)	
	Gel	17 (77)		8 (100)	
	Foam	12 (55)		8 (100)	
	Honeycomb matrix	2 (9)		5 (62)	
	Specialty	0 (0)		6 (75)	
	Memory foam	0 (0)		3 (38)	
**Cushion provision^a^**					
	Self-purchase	10 (45)		3 (38)	
	Facility owned (shared)	22 (100)		8 (100)	
	Seating specialist recommended	10 (45)		3 (38)	

^a^Respondents were allowed to select >1 response in this category on the survey.

^b^In Survey A, participants selected the percentage of residents in their facility for each category.

Survey B responses narrowed in on sitting time for residents at different stages of memory loss, reporting that most residents in their facilities across all stages of memory loss sit for 7 to 12 hours per day (Table 2). Survey B did not distinguish between sitting in a wheelchair and sitting on other surfaces, so 7 to 12 hours can be assumed to represent cumulative sitting time per day.

Both surveys indicated that mobility-related sitting equipment and nonmobile chairs were used in their facilities. Nonmobile sitting equipment was most selected including geriatric chairs and recliners. Wheelchairs were selected by all respondents as available and used in their facilities. The 8 responses in survey B narrowed in on the type of mobility device, and they indicated that in addition to manual wheelchairs, power wheelchairs (n=7, 88%) and scooters (n=4, 50%) were also commonly used in their facilities (Table 3). All (n=8, 100%) respondents of survey B indicated that sitting-related equipment included shared equipment provided by the facility and privately purchased by the resident (n=8, 100%). Survey A responses indicated that more often, mobility devices were privately purchased by the resident (17/22, 77%). Respondents on both surveys indicated that a range of skin protection seat cushion options were provided by the facility, with some residents opting to purchase their own (Table 3). Less than half of respondents endorsed having access to a seating specialist at their facilities in survey A (n=10, 45%) and survey B (n=3, 38%).

Survey B respondents (5/8, 62%) reported a perception that those in more advanced stages of memory loss were more susceptible to sitting-related pressure injuries (Table 4).

**Table 4:** Care provider perceptions about stages of memory loss most susceptible to pressure injuries in long-term care facilities (survey B; n=8).

Stage of memory loss	Respondents, n (%)
Mild	1 (12)
Moderate	2 (25)
Advanced	5 (62)

In survey B with 8 respondents, familiarity with mHealth technologies by care providers was most favorable for self-adjusting surfaces (n=7, 88%) and pressure mapping (n=6, 75%). Perceptions about which type of technology could be most helpful for caregivers were most favorable for self-adjusting surfaces (n=6, 75%) followed by wetness detection (n=4, 50%). Finally, regarding which technology the care provider would want to use if it were available to them, the most favorable response was for monitoring repositioning over time (n=6, 75%) and pressure mapping (n=4, 50%) (Table 5).

**Table 5:** Care provider perceptions about technologies to prevent pressure injuries in skilled nursing facilities (n=8). Respondents were able to select >1 technology type.

Sitting-related technology type	Familiar with technology, n (%)	Helpful for caregivers, n (%)	Would use if available, n (%)
Monitor repositioning over time	0 (0)	2 (25)	6 (75)
Pressure map chair	6 (75)	0 (0)	4 (50)
Self-adjusting surfaces	7 (88)	6 (75)	3 (38)
Timers or reminders	4 (50)	3 (38)	3 (38)
Wetness detection	3 (38)	4 (50)	3 (38)
Alerts or alarms to detect a problem	1 (12)	1 (12)	1 (12)

### Themes From Interviews

The qualitative sample (n=9) included 3 (33%) administrators, 1 (11%) social worker, and 5 (56%) direct care providers (ie, CNAs). Three overarching themes were developed that addressed the study’s research questions.

#### Repositioning Needs Vary Widely in Residents of LTC With AD or ADRD

Participants described a wide range of physical functioning and mobility among residents living with ADRD in their LTC settings. From being “bed-bound,” to using wheelchairs to ambulating unassisted, participants noted a wide range of physical functioning that includes some residents performing activities of daily living on their own and others requiring assistance with transferring, repositioning, toileting, and grooming. One participant noted, “Some of them can get up, but we have to assist them with transferring.” Another participant observed, “Some just want to sit in their [wheel]chair and not be bothered.” Another direct care worker observed, “There are some residents that don’t get up at all.”

In terms of range in time spent sitting per day, a participant shared, “The majority of our residents range kind of in the middle.”

Staff encouraged residents with higher functioning and mobility to engage in physical activities, such as dancing, playing games, and other group activities. For lower-functioning residents, staff were available to assist with transferring, walking, performing activities of daily living, and encouraging residents who were physically able to participate in recreational activities, such as bingo, bowling, and playing cards, to name a few. One participant noted, “We do music over there, so when they’re dancing, they’re...moving and most of them love dancing.” Residents with lower mobility or physical functioning participate in more passive activities, such as watching movies, assembling blocks and puzzles, or matching and folding socks. A participant who was a social worker stated, “Falls are the biggest concern we have for this population.” On the basis of her experience, a direct care worker recounted, “After they fall, they’re scared to move, scared to get up. They don’t want you touching them.” Another direct care worker advised, “We need to stabilize them, just so they do not fall.”

#### Repositioning Is Problematic for the Highest-Risk Residents With AD or ADRD

All 9 participants in this study, most of whom are direct care workers, acknowledged a corporate policy of repositioning residents every 2 hours throughout the day. One participant stated, “We change them as often as we can, cause...sitting in that chair is not feasible for the skin.” A participant, who was an administrator, stated, “Our policy is for staff to make sure they turn...at a minimum of every two hours and more frequently if needed for the resident’s wellbeing.”

Due to the high turnover of direct care workers in the LTC industry, a problem that was exacerbated during the COVID-19 pandemic, performing weight shifts around the clock to reposition residents is an ongoing challenge. In her role as a social worker, one participant made the following observations:

Typically, our goal is to get everyone out of bed every day, and they have the right to refuse. If someone is in bed enough for pressure ulcers, [that is] a constant concern. In speaking for our memory care unit...we have tight staffing...we get everybody out of bed, and the activities and all of that. If someone is in the bed enough for pressure ulcers to be constant concern, they typically are not aware enough to respond to a lot of prompts.

As a preventive, one participant noted, “You have different paddings...and you have to know where to place them.” Most participants in the study indicated they have an adequate staffing pattern in their facilities to ensure weight shifts are being performed on a periodic basis. Examples include the following quotes:

There’s always someone to help them get up.

We have always tried to sustain a higher staffing pattern.

We have staff sufficient to be able to help that.

It should be noted that 2 (22%) participants in the study who provided direct care to residents described feeling overwhelmed, at times, adhering to existing weight shift policies. Direct care workers in the study described daily challenges they encounter when attending to residents with cognitive impairment who need assistance repositioning. The following quotes exemplify the challenges direct care workers face in their efforts to reposition residents with ADRD, particularly wheelchair users:

A lot of my residents don’t want to be touched.

They don’t want that range of motion.

Some of them are very stubborn and they don’t want to do it.

Some of them get a little agitated.

It’s hard to explain to them, because they’re not gonna understand.

You gonna get hit by some of ‘em, because it’s gonna scare them.

Some of them just wanna sit in their chair and not be bothered.

According to our sample of participants, residents with mild cognitive impairment tend to be more cooperative and often will help in repositioning themselves. A direct care worker offered the following example: “You say turn, leg over leg, and they move over.” The chief operating officer of one facility stated, “They may not always understand why, but they understand that we need them to move.” Another participant stated, “We haven’t had any in a long time that refuse to turn for you.”

Overall, participants in the study acknowledged and understood the significance of periodic weight shifts and repositioning and described a breadth of experiences in preventing pressure injuries in their resident populations. Most participants indicated that pressure ulcers were not a major problem in their facilities.

#### Innovative Technologies Can Help and Hinder Care

Residents living with AD or ADRD, who spend most of their waking hours in wheelchairs, require repositioning and frequent weight shifts throughout the day to prevent pressure injuries. In this vein, participants described a variety of techniques they have used to assist residents at risk for developing pressure injuries. For instance, one participant stated she makes “sure they have...good footrest to rest the leg, because mostly the sores [are] in the back sometimes.” Another participant stated, “Sometimes we put a pad or cushion on the chair to make it much softer; sometimes...we put a pad there to kind of ease them off that pressure point.” Another participant, a director of nursing, offered the following insight: “We have a restorative team that...helps with passive range of motion...to get those joints moving.”

In response to the video demos, participants offered a range of opinions on the utility and feasibility of these specific technologies and the generic concepts for use in their facilities. Overall, the participants were impressed with the technologies. “I think this is amazing,” remarked one participant. Another participant noted, “I think that would be an amazing asset to a memory care unit in assisted living.” A CNA participant offered the following insight: “That would be good. Save us from pulling or pushing.”

Due to advanced cognitive impairment in some residents, other participants were a bit skeptical of the technologies’ utility. For instance, 1 (11%) participant concluded, “There are some residents who can participate and benefit from that, [but] it will be a small percentage.”

Another respondent had the following opinion: “I’m not saying this is hard; I just think it’s too much for them. It’s way too coordinated for them.”

Offering a similar opinion, another participant observed, “For our patient population that wouldn’t be effective...they wouldn’t know to do it, from that part of getting up” (referring to the part of the video that shows an alert for user to do a pressure relief).”

Despite the range of opinions, all participants endorsed that, while the sitting-related feedback technologies could be independently managed by a small percentage of residents with higher functioning, the feedback may be too complex for those with more advanced executive function impairment. As a consensus, participants felt that the technologies would be much more suitable for direct care workers (eg, CNAs) to encourage periodic weight shifts. In addition to direct care workers, they responded that sitting-related feedback technologies might also be useful for informal caregivers (eg, family members) to help their loved ones increase in-seat movement.

## Discussion

### Primary Findings

Our findings shed light on unique considerations that may impact the balance between helpful and burdensome when implementing assistive technologies for use by caregivers in LTC facilities with residents who have AD or ADRD, specifically to facilitate repositioning needs during sitting. While caregivers were interested in feedback tools to help them be more efficient or effective in repositioning residents, successful implementation may be challenging due to barriers around available time to learn or use a new technology in their daily workflow. Caregivers also brought awareness to the challenge of repositioning residents with AD or ADRD who experience agitation or combativeness in later stages. These behavioral challenges require a different approach than simply providing reminders that it is time to reposition someone or visual feedback about their pressure distribution. Thus, the assistive technologies for managing sitting-related pressure presented to caregivers in this study only partially address the caregiving needs for people with AD or ADRD in LTC settings.

Due to the ongoing shortage of direct care workers in LTC facilities and 58% of LTC residents having a diagnosis of AD or ADRD, the resulting workload of the currently employed direct care workers and participants increased and they noted that reminders to reposition residents could help with their required monitoring [5,23]. However, even with feedback about pressure or delivery of reminders, completing the required repositioning remains a challenge for already burdened caregivers who, in interviews, acknowledged the need for frequent repositioning of residents but lacked time or resources to do so. Thus, future implementation of feedback technologies will need to carefully consider this barrier, where technology could be perceived as both helpful and futile if the burden of care is not addressed adequately.

Technologies that offer automatic repositioning (eg, self-adjusting cushions [24]) could potentially help reduce caregiver workload by partially managing pressure relief for residents who cannot physically reposition themselves. Furthermore, higher functioning residents with mild memory loss could potentially reposition themselves independently if the feedback technologies are appropriately adapted for their cognitive limitations and if it is safe for the resident to do so [25]. While care providers did not endorse currently using any type of sitting-related technologies, they leaned on a diverse set of strategies for ensuring regular repositioning or activities to increase overall movement among the residents. These strategies were effective with residents with mild cognitive decline who tended to be more cooperative when provided with cues or instructions. However, residents with advanced cognitive decline were more likely to be resistant to movement and, in general, may be difficult to provide care for due to increased agitation, combativeness, and resistance. As a result, residents with more significant cognitive decline may spend more time sitting without pressure relief, resulting in an increased risk of pressure injuries. Understanding the end-user needs is critical for the successful implementation of any new technologies. While we only included a small number of participants and a limited number of care facilities, an important consideration arose—the added impact of cognitive decline as AD or ADRD advances. This creates a layer of complexity around how caregivers approach repositioning residents in LTC.

In the realm of LTC facilities, the integration of technology, particularly within memory care units, holds the potential to enhance both resident well-being and operational efficiency [26]. Various technologies, such as physiological monitoring systems, emergency detection systems, and those designed to foster social interactions, have been used over the years. However, a review study by Chan et al [27] revealed that certain technologies, such as position sensors aimed at reducing falls, have proven ineffective in LTC settings [27]. Despite fall detection systems having a history dating back to the 1970s, their adoption in nursing homes remains varied and limited. Similarly, pet robots and robotic seals, intended to manage behavioral and psychological symptoms of dementia, were found to be ineffective [27]. Technologies designed to promote movement, including the Leaf Patient Monitoring System (Smith+Nephew Leaf* System), M.A.P.TM Continuous Bedside Pressure Mapping System (CBPM; Wellsense), and ForeSite Intelligent Surface (XSensor), have seen limited evaluation in LTC, with Leaf being an exception, demonstrating acceptance [8] and improved turn compliance [28].

Regarding the use of interface pressure map (IPM) feedback as a potential strategy, while nursing staff indicated the pressure feedback was helpful in their efforts to reposition patients, IPM used on beds in the hospital setting did not yield significant changes in pressure injury outcomes in a geriatric or internal medicine ward, as noted by Gunningberg et al [29]. However, when IPM was used by OTs and PTs to evaluate and select seating cushion needs in LTC, the incidence of pressure injuries was reduced [30]. The challenges of implementing technology in LTC environments are evident, and the transition from adoption to implementation to tangible outcomes in pressure injury reduction is particularly arduous. Few products have achieved success, underscoring the importance of involving key stakeholders early in the development process to enhance the likelihood of success [31].

Overall, results from these analyses confirmed and expanded our understanding of the needs of LTC caregivers who care for residents with AD or ADRD in terms of managing sitting-related pressure. Future research could identify how feedback technologies could support residents with mild memory loss to more independently manage pressure and support those with higher acuity, including those admitted with pressure injuries, by offering technologies, such as automated or self-adjusting seat cushions. As the population grows older and caregiver shortages increase across the United States, the use of appropriate technologies may increase effectiveness and efficiency in preventing pressure injuries.

### Limitations

The participants in this study provided consistent feedback for developers of sitting-related technologies; however, the study has a few limitations. Survey response was low and may reflect limited interest in the topic of managing pressure injuries related to sitting in the greater context of other care provided by the participants. It may be a result of staffing barriers following the demands the pandemic placed on direct care providers. Survey response may have improved with remuneration for time to complete the survey. Surveys A and B differed slightly in their presentation of items and level of detail, making it difficult to combine the responses. We did not gather facility-level data, so it is not known how many respondents were from a particular facility, or how many total facilities were represented across respondents. While the purpose for structuring the surveys in this way was to gather preliminary information, future surveys and interviews across larger samples could include a more in-depth exploration within and across facilities because they can differ in culture and policy.

### Implications for Practice

The aging population in the United States is increasing, acuity is increasing, and caregiver availability is decreasing. Innovative technologies could meet this gap by helping caregivers be more effective and efficient in adhering to the repositioning needs of residents in LTC. Preventing pressure injuries in patients with AD or ADRD, particularly those in the more advanced stages, can improve their quality of life. Effective sitting-related feedback technologies may have the potential to reduce the cost burden created by sitting-acquired pressure injuries.

### Conclusions

Our findings confirm that the care providers in the LTC settings we engaged with are interested in sitting-related feedback technologies that can improve their efficiency and effectiveness. While care providers indicated that they understand the clinical guidelines and their facility’s procedures for repositioning and they have access to seating professionals and appropriate pressure-relieving seat cushions, finding time to reposition residents is one of their primary barriers. Thus, future implementation should consider nursing burden, the cost-benefit balance, and the demands of learning and using new tools. Stakeholders’ strongest interest in adopting sitting-related technology was in systems that automate pressure redistribution in some way, show how pressure is distributed on a chair or wheelchair, or provide feedback via reminders that someone needs to be repositioned.

In the LTC context, care providers may prefer technologies that reduce their care burden in some way versus offering them reminders to perform a task that they may not have time to complete, even though they have the knowledge, skills, and access to resources to help them perform the task.

## Appendix

Multimedia Appendix 1 Interview questions, survey A items, and survey B items.

## Abbreviations

AD: Alzheimer disease
ADRD: Alzheimer disease and related dementias
CNA: certified nursing assistant
IPM: interface pressure map
IRB: institutional review board
LTC: long-term care
mHealth: mobile health
OT: occupational therapist
PT: physical therapist

## Data Availability

The datasets generated or analyzed during this study are available from the corresponding author on reasonable request.
