# Supplementary material for: Stakeholder Perspectives on mHealth Technologies to Prevent Sitting-Acquired Pressure Injuries in Long-Term Care Facilities: Mixed Methods Study
Source: JMIR Form Res. 2025 Sep 17;9:e59590. doi: 10.2196/59590 (PMC12489400; doi:10.2196/59590)
Supplement: Multimedia Appendix 1 [file formative_v9i1e59590_app1.docx]

Interview Protocol – Staff

**Introduction and Explanation**

***[Interviewer- Welcome the interviewee. Introduce yourself and notetaker (if applicable). Briefly describe your role in the study. Ask participant to verbally identify themselves (say their FULL name and role), even if you already know who they are.]***

***Example: “Welcome and thanks for meeting with me/us. My name is _________ and I’ll be interviewing you today. I would like to introduce you (insert name) who will be helping with this process, taking notes and recording the session. We are both researchers on the study. Can you share with us your name and your role or job title at this facility?***

***Thank you. Can you also provide us with the best email address for you? We will send you an e-gift card as a thanks for your participation and want to make sure we have your correct email address.”***

Show the participant the study information sheet and let them know you are going to briefly review it with them.

The purpose of this interview is to learn about the use of wheelchairs among your residents with Alzheimer's Disease and Related Dementias. We are also interested in your facility's practices related to caring for these residents. The discussion pertains only to residents with Alzheimer's Disease and Related dementias who use wheelchairs. Your input will inform our study to develop a seat monitoring system to increase movement and prevent pressure ulcers. The interview is scheduled to last 45 minutes to an hour and will include some general and some specific questions about your role and facility’s procedures.

Review any other information from the study information sheet that you think is pertinent.

Before we move forward, what questions do you have for me?

**Questioning**

***[Allow participant to respond to the main question. If they provide short answers or are not willing to elaborate, follow up with some of the prompts listed below each question.]***

*Note. Questions highlighted in blue are the most important questions; should the interview need to be cut short, please focus on these questions.*

**Part 1**

To start, please describe your day-to-day activities in your job here.

Can you talk a bit about the range of functional independence among the residents you care for? In other words, what is the range of your residents’ ability to perform activities of daily living?

Prompt: What is the mobility range of the residents you care for?

Prompt: How do they get around the facility?

Prompt: What type of staff assistance do residents need to transfer into and operate their wheelchairs?

Could you talk about your role in performing weight shifts or making sure residents move in their wheelchairs/chairs?

Prompt: How often would you say you assist with weight shifts?

How involved are residents in the process of performing seated weight shifts?

Prompt: How aware are residents of the need to move regularly to prevent pressure ulcers/bed sores?

Prompt: How much assistance do residents need to move regularly to prevent pressure ulcers/bed sores? For example, do they just need prompts to move and can do it themselves, or do staff need to move them?

Describe the facility’s policy on conducting weight shifts.

Prompt: From your perspective, is this policy generally followed?

Prompt: If so, what helps make this policy easy to follow?

**Part 2**

We are interested in understanding how technology might help with adhering to seated weight shift policy or recommendations to prevent pressure ulcers/sores. What technology do you currently use when caring for residents?

Prompt: For example, do you use smart phones, tablets, pressure mapping, fall detection sensors, turn timers, etc.?

Prompt: If you use tablets, how many are available? How are they used by staff, e.g., one per unit, one per staff member, etc.?

What are your facility’s rules about patient monitoring technology use?

Prompt: If we were to introduce a patient monitoring technology that helped prompt weight shifts, what would we need to know?

We have a video that demonstrates some technology our study partners are interested in. We are going to show that video now, then we will ask you some additional questions related to this technology.

***[Show video 1.0]***

What types of feedback information would you find helpful to improve resident care?

Prompt: e.g., positioning/posture information, regular reminders to perform weight shifts, information about missed weight shifts, positive reinforcement, etc.

Prompt: Would any of the patients benefit from receiving their own feedback vs. caregivers receiving feedback?

What would be the most useful device for receiving patient monitoring feedback regarding weight shifts?

Prompt: E.g., personal phone, dedicated tablet, etc.?

How many and where within the unit?

How many patients per device?

We have another brief video that shows a different way that this technology can be used. After this video, we will ask a few more questions.

***[Show video 2.0]***

We are interested in knowing more about how games could encourage in-seat movement. What do you think about the idea of giving residents a game to encourage in-seat movement?

Prompt: What kind of games/activities do residents engage in now? What might they be interested in?

Prompt: Tell us about the attention span and cognitive ability of the residents you care for.

We would be interested in conducting some kind of usability test as a follow-up study – this might involve testing a technology with, for example, ten residents for a week. Do you think your facility would be interesting in helping us test something like this?

Prompt: Who would we need to contact about arranging something like this?

Prompt: What might we need to consider to get staff/residents to participate?

Thank you. Before we wrap up, I would like to know if there’s anything else we didn’t talk about that is important for us to know about monitoring patient weight shifts.

**Summary and Closure**

Thank you for your input. We appreciate your time and your willingness to share your thoughts and experiences. The interview is now finished, and we will stop the recording. Thank you!

Verify email address for gift card once more.
